# First-person experience and yoga research: studying neural correlates of an intentional practice

**DOI:** 10.3389/fnhum.2015.00085

**Published:** 2015-02-25

**Authors:** Elizaveta Solomonova

**Affiliations:** Individualized Program, University of MontrealMontreal, QC, Canada

**Keywords:** yoga, neurophenomenology, contemplative sciences, meditation, intention, neural correlates

## Introduction

Recent years have seen a dramatic increase in the scientific study of contemplative practices. While seated meditation practices have historically been at the center of inquiry in contemplative sciences, movement-based practices, such as yoga, t'ai chi, qigong, and others, are currently coming to the forefront of this discourse. In her introduction to the present Research Topic, Schmalzl et al. (2014) introduce movement-based contemplative practices (MBCP) and present their essential qualities: MBCPs are embodied and attentive to kinesthetic and proprioceptive sensations; are structured by intentional movement; and are contemplative, that is, characterized by deliberate observation and non-judgmental awareness.

In this opinion paper, I focus on the necessity of studying intentional and experiential aspects of yoga as a MBCP, and on the role of first-person experiential reports in the neurophenomenological investigation of yoga and other MBCPs. I propose that the difference between yoga as a contemplative practice and yoga as a form of physical exercise needs to be assessed through nuanced investigation of subjective experience aimed at illuminating short- and long-term intentions and goals underlying yoga practice as well as dynamic variations within the lived experience of yoga.

## Embodiment and neurophenomenology

Theories of embodiment, such as enaction (Thompson, 2005, 2007; Noe, 2006; Stewart et al., 2010) stress the irreducible foundational links between the mind, the world and the body as conditions of possibility for consciousness. Rooted in the phenomenological tradition of Husserl (1982) and Merleau-Ponty many embodied mind theorists see movement not only as a pragmatic function of interacting with the world, but as a dynamic and plastic way of knowing and as a formative root of one's selfhood and subjectivity (Morris, 2004, 2010). The concept of a “lived body,” derived from Merleau-Ponty (2012), reflects subjectivity conditioned by kinesthetic patterns and bodily habits throughout a lifetime. Yoga practice, in this view, consists of systematic change and of deconditioning of the “lived body” from its earlier habit patterns and creating new patterns and neural connections. Contemporary neurophysiological evidence lends support to the embodied mind approaches, placing sensorimotor “coupling” (Thompson and Varela, 2001) between an individual's moving body and the world at the center of research on subjective experience.

Recent research on contemplative practices, predominantly focused on sitting meditation, has suggested a role of sustained contemplative training for processes of neuroplasticity (Manna et al., 2010), self-awareness (Vago and Silbersweig, 2012) attention modulation, (Lutz et al., 2008b; MacLean et al., 2010), and emotion regulation (Lutz et al., 2008a; Sahdra et al., 2011), among others.

Studying yoga as a MBCP would highlight specific contributions of intentional and dynamic bodily processes to embodied cognition, including processes associated with intentional movement, attention to bodily states, and brain changes linked to variations in the experiential “lived body” and in underlying nervous system due to sustained physical and mental asana practice.

Neurophenomenology (Varela, 1996; Lutz and Thompson, 2003) is the preferred method of inquiry for contemporary contemplative neurosciences and empirical studies of embodied and enactive cognition. The defining feature of neurophenomenology is the use of sophisticated objective neurophysiological measurements in conjunction with nuanced first-person methodologies. Within this framework, objective, and subjective data are seen as mutually constraining and informing, and dynamic methods of examining conscious experience are preferred. Despite a historical distrust of first-person reports by cognitive neuroscientists (Nisbett and Wilson, 1977), recent years have seen an important rise in the use of first-person methodologies both in the form of questionnaires and phenomenologically-informed practices (Chalmers, 1999). Cognitive neurosciences have gradually opened to the integration of systematic analysis of first-person reports (Overgaard et al., 2008), and a number of rigorous approaches to subjective data are now being developed. One methodology, known as “elicitation interview” (Petitmengin, 2006), has been used in a number of studies, including an investigation of epileptic aura (Petitmengin et al., 2006) and the generation of scientific insight (Petitmengin, 2007).

## Yoga as contemplative practice

While the contemporary form of asana sequences of *Hatha Yoga* is relatively recent (Gard et al., 2014), many schools (such as Ashtanga Yoga and Iyengar Yoga) have referred to the ancient text, Patanjali's *Yoga Sutra* (Miller, 1995; White, 2014), as the philosophical source text defining and situating yoga practice on and off the mat. According to the much cited passage from the *Yoga Sutra*, the definition of the yoga practice is: *citta-vrtti-nirodha*, translated as “cessation of the turnings of thought” (Miller, 1995). The goal of yoga, in the traditional sense, can be conceptualized as a “path to freedom” through “graduate unwinding of misconceptions that allows for fresh perceptions” (Miller, 1995); the approach is similar to the traditional goal of sitting meditation practices, i.e., the concept of *enlightenment*. It would follow, then, that in addition to the expected physical benefits of systematic exercise, yoga would have a number of effects on the mind, and that these effects would resemble at least some of the outcomes observed in meditation research.

One line of inquiry in current meditation research concerns the effort to untangle the distinct effects that different meditation practices may have on the brain. Lutz et al. (2008b) have divided meditation practices into two broad categories of “open monitoring” and “focused attention,” and Travis and Shear (2010) have proposed a third category—“automatic self-transcendence.” Josipovic (2010) pointed out that some states cultivated by meditation practices are not currently conceptualized by cognitive neurosciences, so the process of taxonomy and classification needs to proceed in a careful and highly interdisciplinary manner. In *hatha* yoga, such as Ashtanga yoga (Jois, 2010), practice is often structured around focused attention on breathing (*pranayama*) and postures (*asana*), while a non-judgmental attitude and acceptance of one's current psycho-physical state can be characterized, in part, as open monitoring. Furthermore, Ashtanga yoga has been linked to the trait of self-transcendence (Fiori et al., 2014), suggesting that at least one, possibly all (focused attention, open monitoring, and self-transcendence), and perhaps other yet unidentified experiential categories can be applied to neurophenomenological yoga research.

## Intention

An integral part of contemplative practice, intention, has been largely unexplored in contemplative neurosciences. Goals, reasons, and expectations of practitioners need to be taken into consideration in order to elucidate neural correlates of a practice. Intentionality is understood in phenomenology as an “aboutness” of consciousness; consciousness is always a consciousness “of something” (Roy, 1999). Theories of enaction and embodied cognition have incorporated these ideas into a framework of perception and action being oriented, motivated and purposeful within the individual's relationship with the world. In cognitive science, however, intention is often used as a synonym for doing something purposefully, a motivated goal-oriented behavior, with a certain disposition, and expectation. Intentional behavior has been linked to activity in the right posterior temporal superior sulcus network, to the mirror neuron system (Carter et al., 2011), and to the reward system in humans, which involves such structures as ventral striatum (Fliessbach et al., 2007), ventral tegmental area (D'Ardenne et al., 2008), dorsal striatum, putamen, and caudate nucleus (Haruno and Kawato, 2006). What kinds of rewards/expectations can one study in yoga practitioners? Moreover, since contemplative practices, including MBCPs, typically involve long-term commitment, how can one qualify neurophysiological changes before/during/after yoga practice taking into account various possible expectations/rewards associated with an individual practitioner's motivation?

In contemplative practices in general, setting and maintaining of intention plays an important ritualistic and motivational role, especially in the early stages of practice. Within meditation practices, method and intentions vary between stilling focused attention, open monitoring, self-transcendence, and compassion-based training. Recent meditation research has started to unearth some of the neural correlates of intentional contemplative practices. Focused attention meditation recruits attentional networks including insula, anterior cingulate, frontal-parietal regions, and dorsolateral prefrontal cortex (Dickenson et al., 2013). Further, increased functional connectivity between intrinsic and extrinsic networks was reported in practitioners of non-dual awareness (Josipovic, 2014). Finally, recent research on compassion meditation training showed alterations in inferior parietal cortex and DPLFC, networks underlying social cognition and emotion regulation (Weng et al., 2013). Studying neural mechanisms of yoga practice may involve an interaction between processes of intentional reward-oriented behaviors and different kinds of contemplative focus.

However, intention setting in yoga has not been widely studied, despite being an integral part of the practice of some yoga traditions (an opening prayer in Ashtanga yoga being one such example). One phenomenological study of body-based therapeutic practices, including yoga, presented compelling evidence for the role of both long- and short-terms goals and intentions in practitioners and patients, including specific goals of coping with the present situation and general motivations for exploring qualities of embodiment through practice (Mehling et al., 2011).

As a MBCP, yoga shares some of the intentional, motivational and practical elements of meditation. As a form of exercise, it contributes to overall physical health and wellbeing. It is therefore crucial, in order to conduct neurophenomenological research on yoga and to investigate contemplative and intentional dimensions, to factor in various possible short- and long-term intentions and goals that practitioners may set for their practice.

## Neurophenomenology and yoga

Lastly, not only long and short-term intentions, motivations and expectations may have an effect on the neural correlates of yoga practice; one's subjective experience may also undergo a number of cognitive-affective changes during practice. Indeed, temporal dynamics of a meditation experience have been shown to change as a function of attentional and awareness focus in the course of short neurofeedback practices (Garrison et al., 2013a). Since real-time neurofeedback or high-resolution neuroimaging is unlikely during yoga practice, autonomic system measures can be employed in lieu of EEGs or brain scans. For instance, a recent study has used heart rate variability measures as a proxy for affect regulation, along with detailed first-person reports and subjective rating scales to show dynamic changes in attention, affect and subjective experience of calm/activation during a yoga session (Mackenzie et al., 2014). Research potential for including first-person reports in yoga research is further illustrated by another recent study where women suffering from breast cancer were initiated into Iyengar yoga practice; participants underwent pre- and post-study interviews and kept a journal for the duration of the study (Thomas et al., 2014), revealing personal perspectives on qualities of embodiment, posture, and loss.

Studying the neural dynamics of embodied contemplative practices in conjunction with fluctuations in first-person experience would provide invaluable insight to outcome measures, and also to the moment-to-moment changes in practice, experience and intention, which in turn will help elucidate underlying brain mechanisms and contribute to development of interventions adapted to the needs of specific target groups (e.g., Individuals undergoing cancer treatment, depression, anxiety, chronic pain, etc…). The “lived body” is changed by MBCPs, and these changes can be qualitatively explored to investigate both effects of specific MBCPs and structure of embodied subjectivity in general. While investigating the therapeutic effect of yoga in various populations has been the approach of choice, studying healthy individuals involved in sustained contemplative yoga practice (Fiori et al., 2014) may illuminate long-term effects of the practice. This approach has been fruitful in meditation research both by selecting “expert” meditators as participants (Nicholson, 2006; Khalsa et al., 2008; Garrison et al., 2013b), and longitudinally, during a 3-month long Shamatha meditation retreat (MacLean et al., 2010; Saggar et al., 2012; Jacobs et al., 2013). Studying “expert” yoga practitioners can illuminate important aspects of the practice, for instance, some advanced yoga practices emphasize the possibility of awareness without sensory content, such as *purusha* in the yogic tradition (Maehle, 2007). This and other aspects of yoga need to be assessed in a phenomenological way. A neurophenomenological approach is needed in order to assess long- and short-term expectations as well as moment-to moment fluctuations during yoga practice and their neural correlates. Lastly, employing not only expert practitioners but also practicing scientists (Desbordes and Negi, 2013) may contribute to more comprehensive and nuanced contemplative neuroscience of yoga and other MBCPs.

### Conflict of interest statement

The author declares that the research was conducted in the absence of any commercial or financial relationships that could be construed as a potential conflict of interest.
